# Characterization of Arsenic and Atrazine Contaminations in Drinking Water in Iowa: A Public Health Concern

**DOI:** 10.3390/ijerph20075397

**Published:** 2023-04-04

**Authors:** Taehyun Roh, Peter S. K. Knappett, Daikwon Han, Gabriele Ludewig, Kevin M. Kelly, Kai Wang, Peter J. Weyer

**Affiliations:** 1 Department of Epidemiology and Biostatistics, Texas A&M University, College Station, TX 77843, USA; 2 Department of Geology and Geophysics, Texas A&M University, College Station, TX 77843, USA; 3 Interdisciplinary Graduate Program in Human Toxicology, University of Iowa, Iowa City, IA 52242, USA; 4 Department of Occupational and Environmental Health, University of Iowa, Iowa City, IA 52242, USA; 5 Department of Biostatistics, University of Iowa, Iowa City, IA 52242, USA; 6 Center for Health Effects of Environmental Contamination, University of Iowa, Iowa City, IA 52242, USA

**Keywords:** arsenic, atrazine, drinking water, water quality, Iowa

## Abstract

Arsenic and atrazine are two water contaminants of high public health concern in Iowa. The occurrence of arsenic and atrazine in drinking water from Iowa’s private wells and public water systems was investigated over several decades. In this study, the percentages of detection and violation of regulations were compared over region, season, and water source, and factors affecting the detection and concentration of arsenic and atrazine were analyzed using a mixed-effects model. Atrazine contamination in drinking water was found to vary by region, depending on agricultural usage patterns and hydrogeological features. The annual median atrazine levels of all public water systems were below the drinking water standard of 3 ppb in 2001–2014. Around 40% of public water systems contained arsenic at levels > 1 ppb in 2014, with 13.8% containing arsenic at levels of 5–10 ppb and 2.6% exceeding 10 ppb. This unexpected result highlights the ongoing public health threat posed by arsenic in drinking water in Iowa, emphasizing the need for continued monitoring and mitigation efforts to reduce exposure and associated health risks. Additionally, an atrazine metabolite, desethylatrazine, should be monitored to obtain a complete account of atrazine exposure and possible health effects.

## 1. Introduction

Growing public health concerns are related to human exposure to drinking water contaminants and their potential adverse effects on health. Drinking water from both surface and ground waters can become contaminated by natural and anthropogenic contaminants that accumulate in water running off the land surface or infiltrating aquifers through the soil [1]. Water contamination along these general pathways leads to exposure to these contaminants through human consumption of the affected water [2]. Arsenic and atrazine are two water contaminants of high public health concern in Iowa, as they are the most prevalent naturally occurring and anthropogenic water contaminants, respectively.

Arsenic is ranked first on the current priority list of hazardous substances of the US CDC’s Agency for Toxic Substances and Disease Registry (ATSDR), reflecting its toxicity and prevalence [3]. Arsenic naturally occurs in the earth’s crust, and soil and water contamination with arsenic vary considerably in different regions of the world and the US, depending on geological and geochemical conditions [4,5]. In Iowa, the north-central region has young glacial sediments of the Des Moines Lobe, produced during the late Wisconsin age (12,000–16,000 years ago), which have higher concentrations of arsenic [6,7]. Inorganic arsenic (arsenate and arsenite) has been detected in groundwater sources used for drinking and has been associated with adverse health effects, including alteration in the nervous system, hyperkeratosis, cardiovascular disease, diabetes, and cancers of the lung, bladder, and skin [8,9,10,11]. Prenatal exposure to inorganic arsenic in drinking water has been associated with fetal death, preterm birth, low birth weight, and some birth defects [12,13,14]. Some mechanisms proposed to cause arsenic-induced toxicity include oxidative stress, genotoxicity, altered signal transduction, and epigenetic changes [15,16]. The US Environmental Protection Agency (EPA) has set the drinking water standard for arsenic at 10 µg/L, or parts per billion (ppb) [17]. However, this standard applies only to public water systems (PWS), and not to private wells.

Atrazine, a chlortriazine herbicide, is the most widely used herbicide to control weeds in field corn [18]. The European Union banned the use of atrazine in 2004 because of its potential to contaminate wate r [19]. However, atrazine is still widely used in the Midwest Corn Belt, including Iowa in the US, with about 60 to 80 million pounds applied annually between 2010 and 2019, according to the USGS [20]. A US Geological Survey (USGS) study detected atrazine in all surface water samples from eastern Iowa in 1996–98, with a maximum concentration of 100 ppb [21]. The EPA drinking water standard (maximum contaminant limit, or MCL) for atrazine is 3 ppb [22]. Moreover, atrazine degradates, desethylatrazine (DEA), and desisopropylatrazine (DIA), are also frequently detected in drinking water, although they are not currently regulated in the US [23]. Exposure to atrazine is also associated with human health problems such as central nervous system dysfunction, endocrine disruption, and cancers, including non-Hodgkin’s lymphoma, prostate cancer, and stomach cancer [24,25,26,27]. Reproductive and developmental toxicities have also been linked to atrazine exposure, including spontaneous abortion, preterm delivery, and intrauterine growth retardation [28,29,30].

Current regulations and monitoring enforcement differ across countries and regions. As part of the United States, Iowa is subject to the U.S. Environmental Protection Agency’s (EPA) regulations (atrazine at 3 ppb and arsenic at 10 ppb). Different countries and regions have set varying limits for arsenic (e.g., 10 ppb in most countries, 5 ppb in New Jersey and New Hampshire in the US, 25 ppb in Mexico, and 50 ppb in Bangladesh) [31,32,33,34], and atrazine (e.g., 0.1 ppb in the EU, and 5 ppb in Canada) in drinking water [35,36]. As a result, the exposure levels and health risks for residents vary depending on their location. Therefore, it is critical to characterize the unique contamination patterns in each country and region.

This paper described the occurrence of arsenic and atrazine in the drinking water supplies of both PWS and private drinking water wells, and the characteristics of the contamination patterns in the state of Iowa between 2001 and 2014. It also discussed the public health significance of exposure to these contaminants in drinking water.

## 2. Materials and Methods

### 2.1. Study Area

**Figure 1:**
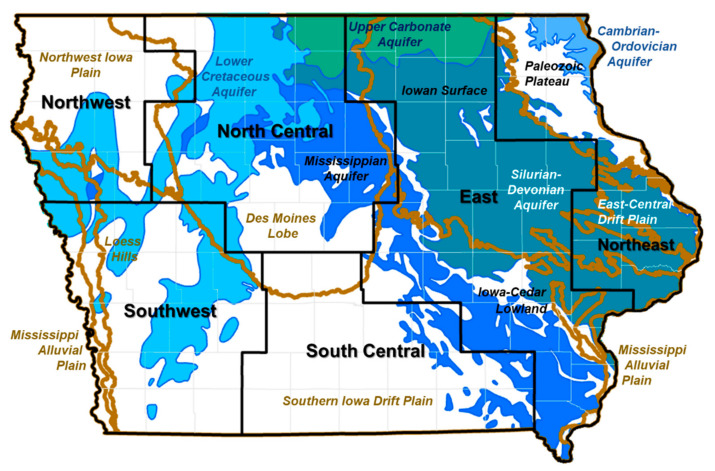
Hydrogeologic regions of the state of Iowa. Iowa was divided into six regions with similar soil type, landscape, and hydrogeologic characteristics as used in Iowa’s state-wide rural well water survey. The bold brown lines on the map represent the boundaries between the regions defined by landforms, and the blue and green colored areas represent the aquifers.

All 99 counties of Iowa were included the study area. Iowa has been divided into six hydrogeologic regions by the Iowa Department of Natural Resources: northeast, east, south-central, southwest, northwest, and north-central (Figure 1) [37]. Each region is distinguished based on similar soil type, landscape, and hydrogeologic characteristics, all of which have the potential to impact the susceptibility of aquifers to contamination. This classification system was applied in the 1988–1989 Iowa Statewide Rural Well Water Survey (SWRL) analysis [37].

The specific characteristics used to define the regions includincludede the depth and age of bedrock, sediment material, aquifer, karst landscape, and thickness and age of glacial drift deposits [37]. In the northeast region, there are shallow Paleozoic carbonate and sandstone aquifers with local karst conditions. The eastern region has a continuous mantle of relatively fine-textured pre-Illinoian glacial deposits over shallow to deep bedrock. The south-central area has Pennsylvanian bedrock with highly variable lithologies and aquifer characteristics, and generally lesser thickness of glacial drift deposits. The southwestern region has deep Pennsylvanian bedrock composed of limestone and sandstone which typically produces little water (low yield), as well as local Cretaceous Dakota sandstone aquifers. The northwestern region has thick glacial drift deposits, and groundwater sources include Cretaceous bedrock units and Dakota sandstone aquifers. The north-central region consists of various bedrocks from Paleozoic carbonate aquifer to Cretaceous Dakota aquifer, and has high relief among major river valleys, with the youngest glacial deposits of the Des Moines lobe. We describe the regional occurrence of arsenic and atrazine in’Iowa's drinking water supplies using these hydrogeologic regions.

### 2.2. Data Sources

This study analyzed four water sources: untreated source water (surface water and public well water), finished public water, and private well water. Untreated public wells were identified from previous USGS reports from the Iowa Ground Water Quality Monitoring Program among all USGS-monitored wells for all purposes [38,39,40]. The 1188 municipal wells included in this study, and their associated data, were retrieved from the USGS National Water Information System (NWIS). The analysis data from 1140 wells for arsenic, 948 wells for atrazine, and 389 wells for DEA and DIA from 1982 to 2009, were included in this study. Data for untreated surface water from 180 sites were also obtained from USGS NWIS. Only data for atrazine were analyzed in untreated surface water since arsenic concentrations were rarely analyzed in surface water samples. In this study, arsenic refers to inorganic arsenic, which includes both trivalent (arsenite) and pentavalent (arsenate) forms. Although arsenite is more toxic than arsenate [41], we were unable to estimate the levels of each species separately due to the unavailability of relevant data.

For public water systems (PWS), data on arsenic and atrazine in 984 PWS in all 99 Iowa counties from 2001 to 2014 were obtained from the Iowa Safe Drinking Water Information System (SDWIS). Monitoring for arsenic and atrazine in PWS is required by the US EPA Safe Drinking Water Act, and water samples were analyzed in laboratories certified by the Iowa Department of Natural Resources (IDNR) using standard methods. The results were submitted directly to the IDNR. The Iowa Administrative Code mandates that one sample should be collected and analyzed once every three years for groundwater systems, and annually for surface water systems or mixed surface water and groundwater systems [42]. If a sample exceeds the MCL, quarterly monitoring is required. Therefore, instead of conducting a monthly comparison, our analysis focused on comparing the seasonal differences.

The SWRL (1988–1989), the Iowa Community Private Well Study (ICPWS, 2002–2003), and the SWRL2 (2006–2008) provided water quality data for Iowa’s private drinking water wells. The SWRL and ICPWS data were obtained from the Center for Health Effects of Environmental Contamination (CHEEC) at the University of Iowa. In the SWRL, water samples were collected from a total of 686 private wells in all 99 Iowa counties and analyzed for coliform bacteria, nitrate, and pesticides. Arsenic testing was not included in the SWRL. In the ICPWS, 236 private wells in 54 Iowa incorporated communities without PWS were sampled and analyzed for pesticides, ammonia, nitrate, arsenic, and bacteria. In the SWRL2, water samples from 473 private wells in 89 counties were analyzed for arsenic, bacteria, nitrate, and other contaminants.

The annual average amounts of atrazine used in 1992–2012 were estimated in kilo-grams per corn acre harvested in each region, based on the data from the US Geological Survey and US Department of Agriculture: 0.46 in the south-central, 0.38 in the south-west, 0.33 in the northeast, 0.31 in the east, 0.19 in the north-central, and 0.13 in the north-west regions [43,44,45].

### 2.3. Water Data Statistical Analysis

Daily median concentrations were calculated from multiple measurements for each day and were used for further statistical analysis. For figures describing trends over the study periods, yearly median concentrations for each sampling site were additionally calculated by taking the median of daily medians for a year. For arsenic and atrazine, concentrations were categorized into three groups: (i) below the limit of detection (LOD), (ii) detected with concentrations higher than the LOD but lower than the MCL, and (iii) detected with concentrations over the MCL. The MCLs for arsenic and atrazine were 10 ppb and 3 ppb, respectively. Measurements for atrazine degradation products desethylatrazine (DEA) and deisopropylatrazine (DIA) were categorized as: (a) below the LOD, and (b) higher than the LOD. To minimize the impact of changing LODs over time, the maximum LODs for each analyte were used (5 ppb for arsenic in public wells, 1 ppb for arsenic in all other sources, 0.2 ppb for atrazine, and 0.1 ppb for DEA and DIA).

To analyze the longitudinal and repeated measures data, we used the PROC MIXED procedure of SAS to fit a mixed-effects model. This model treated each water station or well as a random effect to account for unbalanced data from repeated measurements at the same site [46,47]. The model estimated relationships between region, water source, season, well depth, aquifer type, and the detection and concentrations of contaminants. Categories of arsenic and atrazine concentrations were treated as a continuous variable in the model as they were ordinal. Specifically, a value of 1 was assigned for concentrations lower than the LOD, 2 for concentrations between LOD and regulatory level, and 3 for concentrations greater than regulatory level. The overall significance of association was evaluated using the F value, which compares the difference between categories. For example, the overall significance over regions means that at least one region is significantly different from other regions. If the overall significance was observed, Tukey’s test was performed for post hoc pairwise comparisons. A mixed effects model was also used to estimate correlations among atrazine, DEA, and DIA, with repeated measurements linked over time as suggested by Hamlett et al. [48,49]. SAS 9.4 software was used for these analyses, and results were considered statistically significant if the *p*-value was less than 0.05.

### 2.4. Geographic Mapping

The geodata for boundaries of Iowa and its counties were downloaded from the National Resources Geographic Information System of the Iowa Department of Natural Resources website, and the aquifers and landforms were obtained from the Esri ArcGIS website. The maps were created using ArcGIS Pro 3.0.0 software (Esri, Redlands, CA, USA).

## 3. Results

### 3.1. Occurrence of Arsenic in Public Water Systems

**Figure 2:**
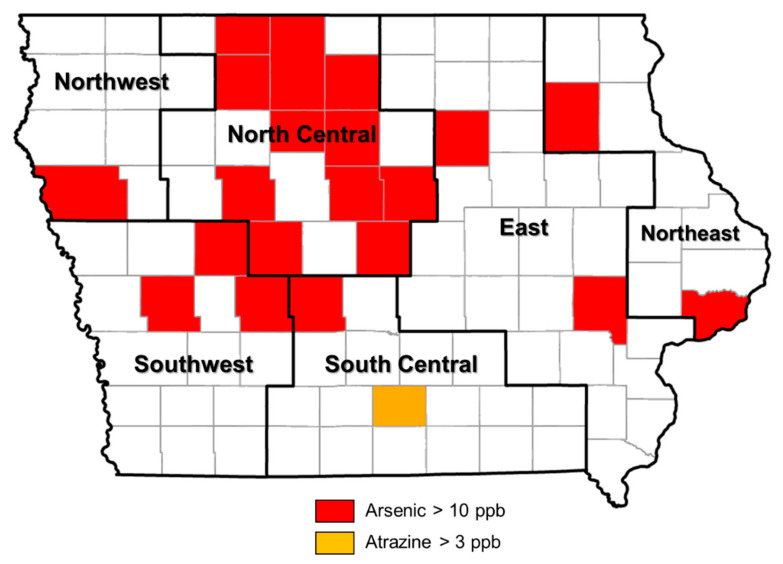
Spatial distribution of counties with levels of arsenic and atrazine exceeding current drinking water MCLs in PWS samples, 2001–2014.

**Figure 3:**
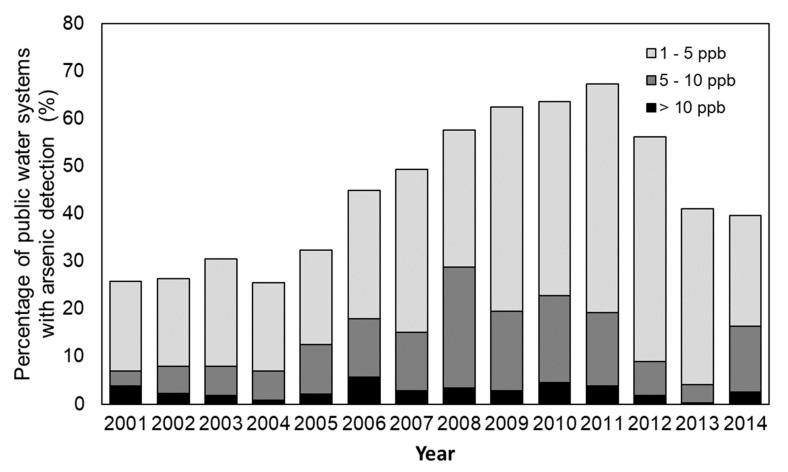
Percentage of public water systems by annual median arsenic category, 2001–2014.

We analyzed the occurrence of arsenic in Iowa’s PWS over a 14-year period (2001–2014) by hydrogeologic region, season, and water source. Arsenic was significantly more prevalent in the north-central and southwestern regions (Table 1). Figure 2 illustrates the consistent spatial distribution of counties with arsenic levels exceeding the current drinking water MCL of 10 ppb. The results showed no significant differences in arsenic detections across seasons and water sources (Table 1). In 2014, over 40% of public water systems had arsenic at levels > 1 ppb, with 13.8% having arsenic at values between 5 and 10 ppb and 2.6% above 10 ppb (Figure 3).

### 3.2. Occurrence of Arsenic in Public Wells

Arsenic was significantly more prevalent in the north-central region (Table 2). Arsenic was detected in a significantly greater number of wells that exceeded 100 ft in depth, compared to wells shallower than this threshold. Arsenic was significantly more prevalent in groundwater samples from glacial drift aquifers compared to all other types of aquifers.

### 3.3. Occurrence of Arsenic in Private Wells

**Figure 4:**
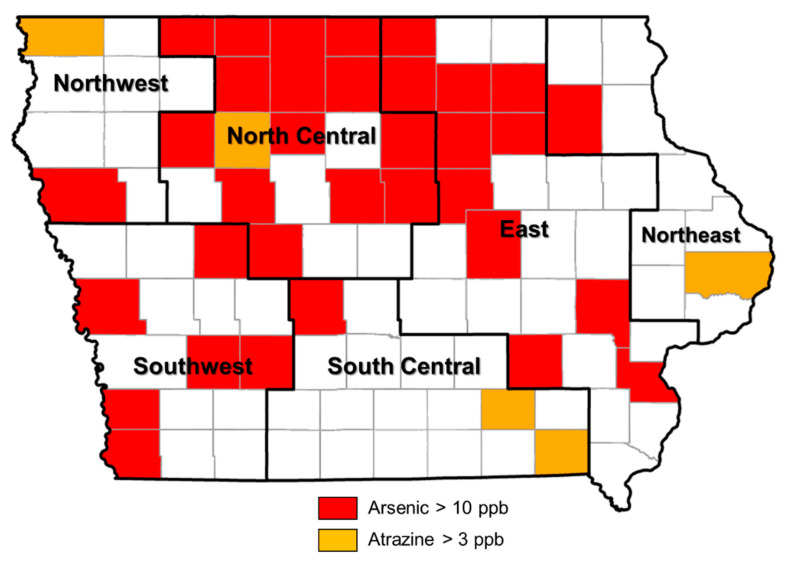
Spatial distribution of counties with levels of arsenic and atrazine exceeding current drinking water MCLs in private well samples, 2001–2014.

In the samples from private wells, arsenic was found to be significantly more prevalent in north-central Iowa compared to other regions (Table 3). In north-central Iowa, 64% of the samples had detectable arsenic; 20% had arsenic concentrations that exceeded the MCL (>10 ppb) (Table 3). The spatial distribution of counties also showed a similar trend (Figure 4). Observed arsenic concentrations were significantly lower in the samples from the shallowest wells.

### 3.4. Occurrence of Atrazine in Public Water System

**Figure 5:**
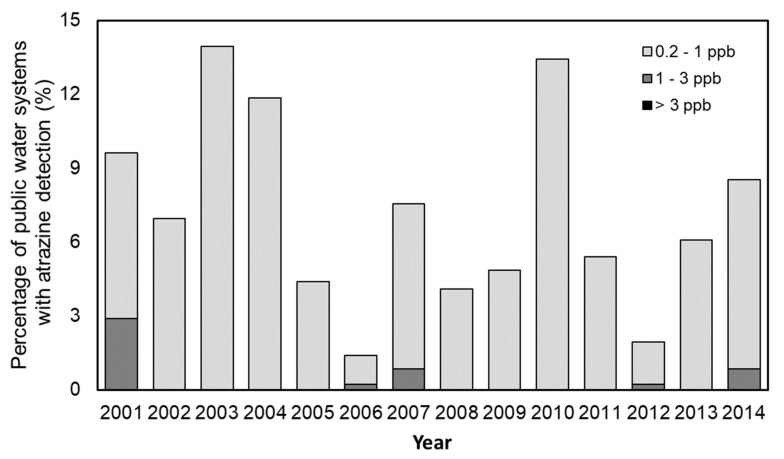
Percentage of public water systems by annual median atrazine category, 2001–2014.

Atrazine detections in PWS were significantly greater in the south-central region compared to other regions (Table 4). The detections of atrazine were significantly higher in the summer, decreasing over the following seasons. Atrazine detections and the frequency of MCL violations were significantly higher in the south-central region, the summer, and surface water-sourced systems. One county in the south-central region had atrazine levels exceeding the current drinking water MCL of 3 ppb between 2001 and 2014 (Figure 2). There were no public water systems with annual median levels of atrazine >3 ppb in the study period (Figure 5). Data on DEA and DIA detections in Iowa’s PWS were not presented, as minimal analyses were conducted (365 samples analyzed for degradates).

### 3.5. Occurrence of Atrazine and its Degradates in Public Wells

In the public wells, Iowa’s northwest and northeast regions had significantly higher atrazine detections (Table 5). Wells deeper than 100 feet had significantly lower atrazine detections and violations. Groundwater samples from the alluvial aquifer showed significantly more prevalent atrazine detections, with 1.1% of samples in violation. DEA and DIA are major degradation products of atrazine. DEA was significantly less detected in groundwater samples from deeper wells with a depth greater than 100 feet (Table 6). In the northwest region, DEA was most frequently detected (12% of samples) (Table 6). DIA detection was much lower than atrazine and DEA, and did not show significant differences over the region, season, and well characteristics (Table 7).

### 3.6. Occurrence of Atrazine and its Degradates in Private Wells

On a regional basis, the south-central region had more samples with concentrations over the MCL, while the north-central region had fewer detections than other regions (Table 8). There were no statistically significant regional and seasonal variations in atrazine concentrations, but it was significantly less prevalent in the deepest wells (>100 ft). Although five counties had atrazine levels exceeding the current drinking water MCL of 3 ppb, the spatial distribution of these counties did not show a specific trend (Figure 4).

There was no significant difference in DEA detection over regions (Table 9). Significantly more detections and concentrations of DEA were observed in the shallow wells (<50 ft). DIA detections were more prevalent in the fall and winter, and significantly less prevalent in the spring (Table 10).

### 3.7. Occurrence of Atrazine in Surface Water

In Iowa’s surface water, the south-central region had significantly more samples with atrazine concentrations over the MCL than other regions (Table 11). Atrazine was significantly more prevalent, and its concentrations were significantly highest in the spring and summer.

## 4. Discussion

Arsenic and atrazine are the most common naturally occurring and anthropogenic water contaminants in Iowa, posing substantial health impacts to the residents. Arsenic, a naturally occurring element, has been frequently detected in Iowa’s groundwater [5]. Arsenic can have harmful effects on human health, including an increased risk of skin, lung, bladder, and liver cancers, as well as cardiovascular disease and diabetes [8,9,10]. Atrazine is a heavily used herbicide in the US corn belt, including Iowa [20]. It can enter groundwater and surface water through runoff and leaching [50,51]. Atrazine exposure has been linked to an increased risk of certain cancers, as well as reproductive and developmental problems [26,27]. In this study, the occurrences of arsenic and atrazine were investigated in water samples from untreated ground and surface source water for PWS, finished public water, and private wells.

Our findings indicate that groundwater in glacially deposited aquifers had a higher prevalence of arsenic detections compared to other aquifer types. Arsenic was detected at a higher prevalence in groundwater from the deepest wells (>100 ft) and was most prevalent in north-central Iowa. The north-central and southwest regions consist of glacial materials in the Des Moines Lobe and the Southern Iowa Drift Plain, which were produced during the late Wisconsin-age (12,000–16,000 years ago) and pre-Illinoian age (18,000 years ago), respectively, and have higher concentrations of arsenic [6,7]. According to data from public and private wells, recently glaciated north-central areas of the state have the highest concentrations of arsenic in the groundwater, followed by the southwest area, as younger glacial deposits are associated with higher arsenic concentrations [6]. In the northern region, where thicker glacial deposits are present, detectable levels of arsenic were most prevalent in deeper wells [6,52].

In PWS, the 10 ppb arsenic MCL was enforced in 2006 [53]. Nevertheless, the percentage of samples with detectable levels of arsenic was about 31%, with 13.8% having arsenic at levels ranging from 5 to 10 ppb and 2.6% exceeding 10 ppb in 2014. The current regulatory level of 10 ppb for arsenic is considered insufficient to adequately protect public health, as indicated by a few states such as New Hampshire and New Jersey establishing more rigorous levels of 5 ppb [31,32]. This implies that residents in Iowa may be exposed to levels of arsenic through public water systems that pose potential health risks. The use of groundwater as a source is a crucial factor contributing to arsenic contamination of public water systems, as groundwater is more susceptible to arsenic contamination. Our analysis showed that the percentage of water systems with groundwater as their source had increased from 82.7% (1986–1999) to 91.3% (2000–2014). Therefore, increased monitoring for arsenic should be considered, especially in the public water systems sourced from groundwater, due to the known toxicity of arsenic from drinking water exposures.

In contrast, atrazine was frequently detected in surface water, as it is applied on agricultural fields as the most highly consumed herbicide. Atrazine was detected more in south-central Iowa during the spring and summer in the surface water and surface water-based public water. Atrazine use in Iowa has been slowly declining since the early 1990s, although use temporarily increased in specific years based on acres of corn planted during the ethanol production boom [54]. Atrazine use has decreased in the US, accompanied by the rapid increase in the use of glyphosate [43,44]. Although glyphosate is currently the most commonly used pesticide in Iowa, it has been detected at the maximum level of 5.49 ppb in Iowa, which is much lower than the current MCL of 700 ppb [55].

In our study, atrazine was detected in 9% of the PWS samples, with only two samples exceeding the MCL of 3 ppb. Atrazine and its metabolite DEA were the most frequently detected herbicides in surface water [56]; atrazine contamination is more prevalent in PWS derived from surface water (53% of the samples) than in PWS derived from groundwater (1.7% of the samples). Analyses of untreated source waters also showed similar results, with atrazine at levels above LOD in 13% of groundwater samples and 92% of surface water samples. Reduced reliance on surface water as a source for the public water system has led to decreased atrazine detection in PWS, as the percentage of public water systems using surface water has decreased from 14.9% (1986–1999) to 7.1% (2000–2014). Over three million pounds of atrazine was used annually in 1992–2012 in Iowa [43,44]. In the treated PWS water derived from surface water, atrazine detections were most prevalent in the south-central region and in late spring and early summer, resulting from atrazine applications and runoff following rainfall events in these seasons [50]. There was a strong correlation between atrazine concentrations in runoff and stream water [57]. Atrazine persists after application to soils, with up to one-third remaining in the upper few cm of the soil for a month within the application areas. This residual atrazine can then run off to streams or leach into groundwater during recharge [51,57]. This is further supported by high rates of atrazine detection in groundwater samples from the alluvial aquifer in late spring and summer, as described in previous studies in Iowa [58,59,60]. Previous studies have shown that atrazine is less frequently detected in winter in groundwater, owing to the hindrance of recharge from less precipitation and soil freezing [61,62]. According to a USGS study of The Mississippi River between Minneapolis and New Orleans, atrazine, DEA, and DIA are the major pesticides transported along the river, with downstream concentrations much higher than upstream levels [63,64,65,66]. The land surfaces in the northern and western regions of Iowa are higher in elevation than other regions, and much of Iowa’s land used for row crop corn drains toward the east and south, eventually flowing into The Mississippi River [67,68]. This has resulted in significantly higher detections of atrazine in the surface water of the south-central region.

Although atrazine is much more of a threat to surface water than groundwater, atrazine was nonetheless detected in Iowa’s private wells, being found in 3% of the well water samples. The atrazine contamination in groundwater was affected by the amount of annual atrazine use and hydrogeologic characteristics. Atrazine detections were more prevalent in northwest and northeast Iowa compared to other regions. The northwest region of Iowa is characterized by alluvial sediments forming shallow unconfined aquifers, while the northeast region has a high risk of groundwater contamination due to the presence of local karst conditions [37]. On the other hand, the north-central region had the lowest detections of atrazine. This is likely because the use of atrazine per corn acre was low, and the aquifers are deeper than 100 feet in this area [37,43,44,45].

Although 10% of atrazine typically remains in the soil after one year, during this time the missing 90% is metabolized to DEA and DIA by bacterial communities in the soil [69,70]. This metabolism is affected by geochemical and physical properties of the soil including pH, moisture, and temperature [69]. The DEA detection rates and concentrations are much higher than the DIA, as the degradation rate of atrazine into DIA is slow compared with DEA [23,71]. These dealkylated metabolites are more soluble than atrazine and their absorption into soil is weaker, implying a strong ability to move and leach into groundwater [57,72,73]. Further atrazine metabolism in groundwater involves chemical degradation processes such as hydrolysis, which can be influenced by specific mineral content and pH [74]. Atrazine may degrade much slower in deep groundwater aquifers due to lower levels of oxygen and microbial activity, and its metabolites, DEA and DIA, can be stable once they enter the saturated zone of the aquifer [75]. On the other hand, surface water contains higher levels of sunlight and microbial activity, leading to additional mechanisms of atrazine degradation such as photolysis and biodegradation [74]. These processes can cause faster breakdown of atrazine in surface water compared to groundwater [74].

In this study, we found that atrazine metabolites were frequently detected in the groundwater. DEA was more prevalent than DIA in both private well water and PWS water, as DEA is the dominant degradation product of atrazine and more stable than DIA [76,77,78]. Only atrazine is regulated currently; neither DEA nor DIA have MCLs. Although the concentrations of degradates are lower, toxicities of DEA and DIA may be larger as the oral 50% lethal dose (LD50) (DEA 1110 mg/kg bodyweight and DIA 1240 mg/kg) in rats is lower than atrazine (1870 mg/kg) [79]. Therefore, DEA and DIA should be considered for monitoring in drinking water supplies.

Our study provides insights into the factors that contribute to higher levels of water contaminant detection and violations of current water quality regulations. By identifying these factors, targeted investigations and interventions can be implemented to improve water quality in affected communities. Effective and cheap monitoring practices are critical for this purpose, and previous studies in Mexico, Bangladesh, and other regions of the US have demonstrated the efficacy of using portable field testing kits to measure arsenic in drinking water [80,81,82]. Adopting this approach on a frequent and periodic basis can help overcome challenges related to seasonality and timing of sampling, and enable the rapid dissemination of results to affected communities. Additionally, incorporating participatory-based monitoring activities, such as citizen scientist engagement, can help increase awareness of regular monitoring, and expand the sampling size in affected communities [83,84].

## 5. Conclusions

This longitudinal study reveals that the levels of arsenic and atrazine contamination in Iowa’s drinking water vary depending on multiple factors such as source, region, hydrogeology, and human activity. These findings indicate that some residents may be at a greater risk of health issues due to potential disparities in the quality of their drinking water. Protecting water supply sources from pesticides, including atrazine, can be achieved through several methods, such as integrated pest management, establishing buffer zones, adopting conservation practices, and proper land use management [85,86,87]. Implementing these protective measures can help reduce the risk of pesticides entering drinking water supplies. Thorough water quality tests should be conducted before adding new water sources, and filtration systems such as ion exchange, ultrafiltration, and reverse osmosis should be used to remove these contaminants from drinking water to minimize exposure [88,89]. It is also crucial to monitor and regulate the major atrazine degradates due to their high detection rates and toxicity. This study highlights the need for further research on the causes of increased drinking water contamination and the necessity for local, regional, and federal public health departments to closely monitor and mitigate the trends in drinking water contamination.

## Figures and Tables

**Table 1:** Occurrence of Arsenic in Public Water Systems (Drinking Water MCL = 10 ppb).

Categories		Site No.	Samples No.	<1 ppb No. (%)	1–10 ppb No. (%)	>10 ppb No. (%)	Maximum (ppb)
Region	E	286	1002	783 (78.1)	155 (15.5)	64 (6.4)	73.0
	NC *	167	797	321 (40.3)	415 (52.3)	61 (7.7)	66.0
	NE	172	496	388 (78.2)	103 (20.8)	5 (1.0)	14.0
	NW	59	161	121 (75.2)	37 (23.0)	3 (1.9)	47.0
	SC	46	159	107 (67.3)	51 (32.1)	1 (0.6)	12.4
	SW *	87	347	171 (49.3)	162 (46.7)	14 (4.0)	83.0
Season	Spring	454	910	622 (68.4)	241 (26.5)	47 (5.2)	83
	Summer	391	750	472 (62.9)	248 (33.1)	30 (4.0)	66
	Fall	311	620	334 (53.9)	243 (39.2)	43 (6.9)	73
	Winter	352	682	463 (67.9)	191 (28.0)	28 (4.1)	47
Source	Ground	778	2673	1658 (62.0)	868 (32.5)	147 (5.5)	83
	Mix	8	171	160 (93.6)	10 (5.9)	1 (0.6)	17
	Surface	31	118	73 (61.9)	45 (38.1)	0 (0)	4

* Significantly higher prevalence than other categories at *p* < 0.05, based on post hoc tests on linear mixed effect regression.

**Table 2:** Occurrence of Arsenic in Public Wells (Drinking Water MCL = 10 ppb).

Categories		Site No.	Samples No.	<5 ppb No. (%)	5–10 ppb No. (%)	>10 ppb No. (%)	Maximum (ppb)
Region	E	350	489	475 (97.1)	10 (2.0)	4 (0.8)	20
	NC *	252	328	278 (84.8)	24 (7.3)	26 (7.9)	110
	NE	116	146	139 (95.2)	3 (2.1)	4 (2.7)	21
	NW	120	164	152 (92.7)	9 (5.5)	3 (1.8)	22
	SC	82	101	96 (95.1)	4 (4.0)	1 (1.0)	30
	SW	220	310	283 (91.3)	14 (4.5)	13 (4.2)	125
Season	Spring	258	281	266 (94.7)	5 (1.8)	10 (3.6)	116
	Summer	581	698	658 (92.4)	32 (4.5)	22 (3.1)	110
	Fall	385	410	372 (90.7)	23 (5.6)	15 (3.7)	125
	Winter	154	159	150 (94.3)	4 (2.5)	5 (3.1)	20
Well depth	< 50 ft	280	423	396 (93.6)	20 (4.7)	7 (1.7)	90
	50–100	193	289	273 (95.5)	7 (2.5)	6 (2.1)	20
	>100 *	664	841	766 (91.1)	36 (4.3)	39 (4.6)	125
Aquifer	Alluvial	395	592	555 (93.8)	24 (4.1)	13 (2.2)	90
	Bedrock	552	685	637 (93.0)	25 (3.7)	23 (3.4)	125
	Glacial *	185	265	235 (88.7)	14 (5.3)	16 (6.0)	90

* Significantly higher prevalence than other categories at *p* < 0.05, based on post hoc tests on linear mixed effect regression.

**Table 3:** Occurrence of Arsenic in Private Wells (Drinking Water MCL = 10 ppb).

Categories		Site No.	Samples No.	<1 ppb No. (%)	1–10 ppb No. (%)	>10 ppb No. (%)	Maximum (ppb)
Region	E	490	490	328 (66.9)	147 (30.0)	15 (3.1)	160
	NC *	183	184	67 (36.4)	82 (44.6)	35 (19.0)	130
	NE	236	236	193 (81.8)	39 (16.5)	4 (1.7)	17
	NW	43	43	20 (46.5)	22 (51.2)	1 (2.3)	22
	SC	43	43	28 (65.1)	14 (32.6)	1 (2.3)	13
	SW	200	200	89 (44.5)	100 (50.0)	11 (5.5)	74
Season	Spring	90	90	56 (62.2)	29 (32.2)	5 (5.6)	40
	Summer	191	191	100 (52.4)	81(42.4)	10 (5.2)	44
	Fall	318	318	198 (62.3)	98 (30.8)	22 (6.9)	160
	Winter	110	110	73 (66.4)	28 (25.5)	9 (8.2)	130
Well depth	< 50 ft †	112	113	64 (56.6)	45 (39.8)	4 (3.5)	40
	50–100	91	91	25 (27.5)	54 (59.3)	12 (13.2)	160
	>100	179	179	43 (24.0)	112 (62.6)	24 (13.4)	110
Well age	< 1991	282	282	90 (31.9)	161 (57.1)	31 (11.0)	160
	≥ 1991	85	85	31 (36.5)	47 (55.3)	7 (8.2)	28

* Significantly higher prevalence than other categories at *p* < 0.05, based on post hoc tests on linear mixed effect regression; **†** Significantly lower prevalence than other categories at *p* < 0.05, based on post hoc tests on linear mixed effect regression.

**Table 4:** Occurrence of Atrazine in Public Water Systems (Drinking Water MCL = 3 ppb).

Categories		Site No.	Samples No.	<0.2 ppb No. (%)	0.2–3 ppb No. (%)	>3 ppb No. (%)	Maximum (ppb)
Region	E	292	976	925 (94.8)	51 (5.2)	0	2.2
	NC	164	377	374 (99.2)	3 (0.8)	0	0.8
	NE	179	523	496 (94.8)	27 (5.2)	0	1.85
	NW	59	129	126 (97.7)	3 (2.3)	0	0.6
	SC *	47	291	154 (52.9)	135 (46.4)	2 (0.7)	5.57
	SW	90	229	221 (96.5)	8 (3.5)	0	0.4
Season	Spring	357	652	606 (92.9)	46 (7.1)	0	2.2
	Summer *	377	734	646 (88.0)	86 (11.7)	2 (0.3)	5.57
	Fall	289	565	520 (92.0)	45 (8.0)	0	1.76
	Winter	290	574	524 (91.3)	50 (8.7)	0	1.57
Source	Ground	791	2043	2008 (98.3)	35 (1.7)	0	1.85
	Mix	8	176	144 (81.8)	32 (18.2)	0	1.9
	Surface *	32	306	144 (47.1)	160 (52.3)	2 (0.7)	5.57

* Significantly higher prevalence than other categories at *p* < 0.05, based on post hoc tests on linear mixed effect regression.

**Table 5:** Occurrence of Atrazine in Public Wells (Drinking Water MCL = 3 ppb).

Categories		Site No.	Samples No.	<0.2 ppb No. (%)	0.2–3 ppb No. (%)	>3 ppb No. (%)	Maximum (ppb)
Region	E	255	687	585 (85.2)	99 (14.4)	3 (0.4)	5.7
	NC	203	435	403 (92.6)	31 (7.1)	1 (0.2)	3.7
	NE *	98	253	211 (83.4)	39 (15.4)	3 (1.2)	14.3
	NW *	111	355	275 (77.5)	73 (20.6)	7 (2.0)	21
	SC	65	183	163 (89.1)	20 (10.9)	0	1.6
	SW	229	707	637 (90.1)	65 (9.2)	5 (0.7)	7.1
Season	Spring	152	206	138 (67.0)	61 (29.6)	7 (3.4)	13
	Summer	834	2257	1800 (79.8)	438 (19.4)	19 (0.8)	25.5
	Fall	340	578	438 (75.8)	135 (23.4)	5 (0.9)	14
	Winter	53	60	53 (83.3)	6 (10.0)	1 (1.7)	14.3
Well depth	<50 ft	320	986	819 (83.1)	154 (15.6)	13 (1.3)	21
	50–100	201	623	507 (81.4)	111 (17.8)	5 (0.8)	12.8
	>100 †	473	1068	999 (93.5)	68 (6.4)	1 (0.1)	4.3
Aquifer	Alluvial *	380	1339	1110 (82.9)	214 (16.0)	15 (1.1)	21
	Bedrock	365	782	703 (89.9)	78 (10.0)	1 (0.1)	4.3
	Glacial	208	511	479 (93.7)	32 (6.3)	0 (0)	2.3

* Significantly higher prevalence than other categories at *p* < 0.05, based on post hoc tests on linear mixed effect regression; **†** Significantly lower prevalence than other categories at *p* < 0.05, based on post hoc tests on linear mixed effect regression.

**Table 6:** Occurrence of DEA in Public Wells.

Categories		Site No.	Samples No.	<0.1 ppb No. (%)	≥0.1 ppb No. (%)	Maximum (ppb)
Region	E	103	322	293 (91.0)	29 (9.0)	0.31
	NC	81	193	185 (95.9)	8 (4.2)	0.32
	NE	46	137	129 (94.2)	8 (5.8)	0.16
	NW	57	158	139 (88.0)	19 (12.0)	0.24
	SC	30	75	74 (98.7)	1 (1.3)	0.16
	SW	64	211	206 (97.6)	5 (2.4)	0.15
Season	Summer	317	999	938 (93.9)	61 (6.1)	0.32
	Fall	112	119	109 (91.6)	10 (8.4)	0.31
Well depth	<50 ft	71	346	319 (92.2)	27 (7.8)	0.32
	50–100	71	228	205 (89.9)	23 (10.1)	0.31
	>100 †	228	537	517 (96.3)	20 (3.7)	0.26
Aquifer	Alluvial	121	497	456 (91.8)	41 (8.3)	0.32
	Bedrock	177	398	372 (93.2)	26 (6.5)	0.31
	Glacial	83	208	205 (98.6)	3 (1.4)	0.1

**†** Significantly lower prevalence than other categories at *p* < 0.05, based on post hoc tests on linear mixed effect regression.

**Table 7:** Occurrence of DIA in Public Wells.

Categories		Site No.	Samples No.	<0.1 ppb No. (%)	≥0.1 ppb No. (%)	Maximum (ppb)
Region	E	103	322	320 (99.4)	2 (0.6)	0.1
	NC	81	193	184 (95.3)	9 (4.7)	0.3
	NE	46	137	137 (100)	0	-
	NW	57	158	157 (99.4)	1 (0.6)	0.18
	SC	30	75	75 (100)	0	-
	SW	64	211	209 (99.1)	2 (1.0)	0.1
Season	Summer	317	909	897 (98.7)	12 (1.3)	0.3
	Fall	112	112	111 (99.1)	1 (0.9)	0.2
Well depth	<50 ft	90	346	335 (96.8)	11 (3.2)	0.3
	50–100	71	228	225 (98.7)	3 (1.3)	0.2
	>100	228	537	536 (99.8)	1 (0.2)	0.1
Aquifer	Alluvial	121	497	483 (97.2)	14 (2.8)	0.3
	Bedrock	177	398	397 (99.8)	1 (0.3)	0.1
	Glacial	83	208	208 (100)	0	-

**Table 8:** Occurrence of Atrazine in Private Wells (Drinking Water MCL = 3 ppb).

Categories		Site No.	Samples No.	<0.2 ppb No. (%)	0.2–3 ppb No. (%)	>3 ppb No. (%)	Maximum (ppb)
Region	E	457	517	499 (96.5)	18 (3.5)	0	2.3
	NC	203	248	246 (99.2)	1 (0.4)	1 (0.4)	3.4
	NE	215	249	242 (97.2)	6 (2.4)	1 (0.4)	3.2
	NW	79	95	91 (95.8)	4 (4.2)	0	1.7
	SC	164	185	184 (97.8)	2 (1.1)	2 (1.1)	6.6
	SW	158	182	178 (97.8)	4 (2.2)	0	1.1
Season	Spring	312	322	316 (98.1)	5 (1.6)	1 (0.3)	6.6
	Summer	397	410	403 (98.3)	6 (1.5)	1 (0.2)	3.2
	Fall	459	464	452 (97.4)	11 (2.4)	1 (0.2)	4.7
	Winter	278	280	266 (95.0)	13 (4.6)	1 (0.4)	3.4
Well depth	< 50 ft	235	277	262 (94.6)	14 (5.1)	1 (0.4)	6.6
	50–100	184	229	219 (95.6)	8 (3.5)	2 (0.9)	3.4
	> 100 †	337	432	426 (98.6)	6 (1.4)	0	0.4
Well age	< 1991	671	817	797 (97.6)	18 (2.2)	2 (0.2)	3.4
	≥ 1991	78	78	75 (96.2)	3 (3.9)	0 (0.0)	0.8

**†** Significantly lower prevalence than other categories at *p* < 0.05, based on post hoc tests on linear mixed effect regression.

**Table 9:** Occurrence of DEA in Private Wells.

Categories		Site No.	Samples No.	<0.1 ppb No. (%)	≥0.1 ppb No. (%)	Maximum (ppb)
Region	E	457	558	510 (91.4)	48 (8.6)	0.86
	NC	203	272	268 (98.5)	4 (1.5)	1.3
	NE	215	259	235 (90.7)	24 (9.3)	0.42
	NW	79	115	104 (90.4)	11 (9.6)	2.86
	SC	164	202	196 (97.0)	6 (3.0)	0.72
	SW	158	204	200 (98.0)	4 (1.9)	0.24
Season	Spring	374	386	376 (97.4)	10 (2.6)	2.86
	Summer	399	412	389 (94.4)	23 (5.6)	0.64
	Fall	521	532	484 (91.0)	48 (9.0)	0.72
	Winter	278	280	264 (94.3)	16 (5.7)	1.3
Well depth	<50 ft *	235	309	268 (86.7)	41 (13.3)	2.86
	50–100	184	262	252 (96.2)	10 (3.8)	1.3
	>100	337	483	470 (97.3)	13 (2.7)	0.3
Well age	<1991	671	902	850 (94.2)	52 (5.8)	2.79
	≥1991	78	78	69 (88.5)	9 (11.5)	0.28

* Significantly higher prevalence than other categories at *p* < 0.05, based on post hoc tests on linear mixed effect regression.

**Table 10:** Occurrence of DIA in Private Wells.

Categories		Site No.	Samples No.	<0.1 ppb No. (%)	≥0.1 ppb No. (%)	Maximum (ppb)
Region	E	457	517	501 (96.9)	16 (3.1)	1.38
	NC	201	248	241 (97.2)	7 (2.8)	0.92
	NE	215	249	245 (98.4)	4 (1.6)	0.49
	NW	79	95	90 (94.7)	5 (5.3)	3.54
	SC	164	185	181 (97.8)	4 (2.2)	3.1
	SW	158	182	177 (97.3)	5 (2.8)	0.2
Season	Spring	374	386	385 (99.7)	1 (0.3)	3.54
	Summer	399	412	401 (97.3)	11 (2.7)	0.67
	Fall *	521	532	514 (96.6)	18 (3.4)	3.1
	Winter *	278	280	265 (94.6)	15 (5.4)	1.38
Well depth	<50 ft	235	277	263 (95.0)	14 (5.1)	3.54
	50–100	184	229	222 (96.9)	7 (3.1)	3.1
	>100	337	432	419 (97.0)	13 (3.0)	1.38
Well age	<1991	671	817	793 (97.1)	24 (2.9)	3.1
	≥1991	78	78	76 (97.4)	2 (2.6)	0.26

* Significantly higher prevalence than other categories at *p* < 0.05, based on post hoc tests on linear mixed effect regression.

**Table 11:** Occurrence of Atrazine in Surface Water (Drinking Water MCL = 3 ppb).

Categories		Site No.	Samples No.	<0.1 ppb No. (%)	0.1–3 ppb No. (%)	≥3 ppb No. (%)	Maximum (ppb)
Region	E	61	113	8 (7.1)	101 (89.4)	4 (3.5)	5.8
	NC	13	19	4 (21.1)	15 (79.0)	0	0.5
	NE	44	75	9 (12.0)	66 (88.0)	0	2.5
	NW	1	1	0	1 (100)	0	1.6
	SC *	46	74	3 (4.1)	65 (87.8)	6 (8.1)	12
	SW	15	26	1 (3.85)	24 (92.3)	1 (3.9)	4.9
Season	Spring *	73	127	6 (4.7)	111 (87.4)	10 (7.9)	11.2
	Summer *	107	169	15 (8.9)	132 (78.1)	22 (13.0)	25
	Fall	35	82	8 (9.8)	74 (90.2)	0	1.9
	Winter	72	118	8 (6.8)	109 (92.4)	1 (0.9)	3.6

* Significantly higher prevalence than other categories at *p* < 0.05, based on post hoc tests on linear mixed effect regression.

## Data Availability

The data generated and/or analyzed during the current study are available from the corresponding author on reasonable request.
